# European Cancer Prevention Organization on cancer screening: an expert panel position paper

**DOI:** 10.1097/CEJ.0000000000001025

**Published:** 2026-07-29

**Authors:** Luca Bertolaccini, Carlo La Vecchia, Francesco Sardanelli, Mãrcis Leja, Adriana Albini, Cristina Trovato, Giorgio Bogani, Francesca Magnoni, Francesco Di Bello, Franco Roviello, Viviana Galimberti, Fátima Carneiro, Rachel van der Post, Gabriella Pravettoni, Lucia Mangone, Carmen Criscitiello, Mariachiara Negrelli, Matteo Lazzeroni, Vincenzo Nicastro, Lorenzo Spaggiari, Meric A. Altinoz, Jaak Ph Janssens, Giovanni Corso

**Affiliations:** a Department of Oncology and Hemato-Oncology, University of Milan; b Department of Thoracic Surgery, IEO, European Institute of Oncology IRCCS; c Department of Clinical Medicine and Community Health, University of Milan; d Lega Italiana per la Lotta contro i Tumori (LILT) Milano Monza Brianza, Milan, Italy; e Institute of Clinical and Preventive Medicine, University of Latvia, Riga, Latvia; f Scientific Directorate; g Division of Endoscopy, IEO, European Institute of Oncology IRCCS; h Division of Gynecologic Oncology, Department of Surgery, Fondazione IRCCS Istituto Nazionale dei Tumori di Milano; i Division of Breast Surgery, IEO, European Institute of Oncology IRCCS, Milan; j Department of Urology, Fundació Puigvert, Barcelona, Spain; k Department of Neurosciences, Science of Reproduction and Odontostomatology, University of Naples Federico II, Naples; l Department of Oncology, Unit of General Surgery and Surgical Oncology, University Hospital of Siena; m Department of Medicine, Surgery and Neurosciences, University of Siena, Siena, Italy; n i3S – Instituto de Investigação e Inovação em Saúde, Universidade do Porto; IPATIMUP – Institute of Molecular Pathology and Immunology, University of Porto; o Department of Pathology, Faculty of Medicine, University of Porto (FMUP); p Department of Pathology, Unidade Local de Saúde (ULS) de São João, Porto, Portugal; q Department of Pathology, Radboud University Medical Center, Nijmegen, The Netherlands; r Applied Research Division for Cognitive and Psychological Science, IEO, European Institute of Oncology, IRCCS, Milan; s Epidemiology Unit, Azienda USL-IRCCS di Reggio Emilia, Reggio Emilia; t Division of Senology, Humanitas Research Hospital; u Humanitas University, Breast Oncology Unit, Humanitas Research Hospital; v Division of Pathology; w Division of Cancer Prevention and Genetics, IEO, European Institute of Oncology IRCCS; x Mininvasive Oncologic Surgery Department, ASST Grande Ospedale Metropolitano Niguarda, Milan, Italy; y Department of Medical Biochemistry, Acibadem University, Istanbul, Turkey; z European Cancer Prevention Organization (ECP), Milan, Italy

**Keywords:** breast cancer, cancer screening, *Helicobacter pylori*, human papilloma virus testing, liquid biopsy, low-dose computed tomography, lung cancer, population-based programs, precision prevention, prostate-specific antigen testing

## Abstract

**Background:**

Cancer screening is a cornerstone of cancer control in Europe yet marked disparities persist in access, implementation, and quality assurance among national or regional programs. Building on the EU Council’s 2022 recommendations and the European Union’s Beating Cancer Plan, the European Consensus Project (ECP) was established to provide harmonized, evidence-based recommendations for major cancer screening programs and to promote a shift toward risk-adapted, technology-enabled, and equitable prevention models.

**Methods:**

A structured expert panel meeting was convened under the auspices of the European Cancer Prevention Organization, integrating systematic literature review and multidisciplinary discussion to formulate validated, evidence-based recommendations. For each primary cancer site (breast, lung, colo-rectum, cervix, prostate, and stomach), systematic literature reviews identified evidence on reductions in mortality, stage distribution, participation, cost-effectiveness, and organizational quality.

**Results:**

The consensus reaffirmed the evidence of mortality reduction with organized breast, lung, colorectal, and cervical screening, endorsed structured risk-adapted prostate-specific antigen testing for prostate cancer, and recommended *Helicobacter pylori* ‘screen-and-treat’ strategies for gastric cancer in high-incidence regions. Insights into future perspectives of liquid biopsy approaches are outlined.

**Conclusion:**

The ECP Consensus outlines a European roadmap for precision screening, grounded in quality assurance, individual risk stratification, artificial intelligence integration, and public health literacy. These recommendations align with the EU Mission on Cancer and Europe’s Beating Cancer Plan, providing a harmonized roadmap toward precision prevention and equitable implementation of screening across Europe.

## Introduction

Cancer screening is a cornerstone of modern public health strategies, enabling early detection of malignancies and improving survival through timely intervention. Over the past three decades, population-based screening programs have been shown to reduce mortality for several cancer types, particularly breast, cervical, and colorectal cancers. The WHO and the European Commission have both emphasized that effective screening programs must balance benefits and harms, ensuring cost-effectiveness, accessibility, and equity across populations (World Health Organization, 2022).

Despite major advances, appreciable disparities persist in participation rates, access to high-quality imaging or molecular testing, and integration of individualized risk profiles (Santucci *et al*., 2026). The high incidence of lung and prostate cancers, coupled with emerging evidence supporting low-dose computed tomography (LDCT) and prostate-specific antigen (PSA) testing in selected high-risk populations, calls for updated, evidence-based consensus recommendations (de Koning *et al*., 2020; Franlund *et al*., 2022). Meanwhile, precision prevention approaches (leveraging genetic, lifestyle, and environmental risk factors) offer new opportunities to refine screening intervals and modalities (Albini *et al.*, 2025).

In breast cancer, advances in digital mammography and tomosynthesis have enhanced sensitivity but not specificity (Esserman *et al*., 2026); in addition, underdiagnosis (i.e. interval cancer rate), overdiagnosis, and false positives remain concerns that necessitate optimized age thresholds and screening frequency as well as the use of other modalities such as MRI in women at high genetic risk (Saslow *et al*., 2007), in those with a strong family history, and in selected women with extremely dense breasts (Bakker *et al*., 2019).

For lung cancer, randomized trials demonstrated a 20–25% reduction in disease-specific mortality with LDCT screening in high-risk smokers, prompting guideline formulation across Europe and North America (Aberle *et al*., 2011; de Koning *et al*., 2020).

Colorectal cancer screening has been shown to reduce not only mortality but also disease incidence. Organized programs typically rely on fecal immunochemical testing (FIT) with colonoscopy as a confirmatory diagnostic procedure, strategies supported by randomized trials demonstrating improved detection of early stage disease compared with usual care (Atkin *et al*., 2010; Shaukat *et al*., 2013; Zorzi and Urso, 2023; Han *et al.*, 2024; Knudsen *et al*., 2025; Westerberg *et al*., 2026).

Cervical cancer screening, driven by cytology and human papilloma virus (HPV)-based testing, continues to show remarkable efficacy, and the introduction of HPV vaccination further strengthens the preventive continuum (Joura *et al*., 2015; Kyrgiou *et al*., 2020).

Gastric cancer screening, though not widely adopted across Europe, has shown promising results in high-incidence regions, where organized endoscopic programs have demonstrated measurable population-level effects on incidence trends and healthcare demand following implementation (Leja *et al.*, 2014; Sun *et al*., 2026).

However, Europe continues to face persistent fragmentation in the delivery of organized screening programs. The 2022 EU Council Recommendation on Cancer Screening explicitly called for expanding and harmonizing programs for breast, cervical, colorectal, lung, and prostate cancers (European Council, 2022).

The present European Cancer Prevention Organization (ECPO) initiative was conceived in direct response to this policy mandate, aiming to unify scientific evidence and public health action.

In this evolving context, a growing consensus is emerging that future screening must shift from population-level uniformity toward risk-stratified, precision-driven models that integrate artificial intelligence, molecular biomarkers, and personalized risk prediction tools to optimize screening performance and resource allocation (Corso *et al.*, 2023; Albini *et al.*, 2025; Lynge *et al*., 2026).

The ECPO aimed to develop an integrated, up-to-date framework for cancer screening across breast, lung, colorectal, cervical, prostate, and gastric cancers. The objectives were:

1. To summarize the current state of evidence and guideline recommendations for major cancer screening programs in Europe.
2. To identify unmet needs and evidence gaps regarding age ranges, screening intervals, and inclusion of high-risk subgroups.
3. To propose innovative, multidisciplinary, and risk-adapted strategies, supported by technological and educational interventions, to optimize screening participation and effectiveness.

Through this collaborative effort, the European Consensus Project (ECP) aims to align scientific evidence, clinical practice, and public health policy, thereby paving the way for a European model of precision screening that balances efficacy, equity, and sustainability.

## Material and methods

### Study design and scope

The present work represents the outcome of a structured expert panel meeting held under the auspices of the ECP. The initiative was conceived to update and structure evidence-based recommendations for cancer screening of six major malignancies: breast, lung, colorectal, cervical, prostate, and gastric cancer. The consensus was reached through a multidisciplinary expert discussion integrating a systematic literature review, critical appraisal, and iterative refinement of recommendations. No formal Delphi or RAND/UCLA scoring system was applied; instead, agreement was achieved through structured discussion among participants.

### Participants and structure

Panelists included oncologists, surgeons, epidemiologists, pathologists, radiologists, gastroenterologists, public health specialists, biochemists, and molecular biologists from multiple European centers. Participants were selected to ensure balanced representation of disciplines, gender, and geographical regions within the European Union. All contributors declared potential conflicts of interest before participation, and the process was conducted independently of any commercial sponsorship.

The expert meeting followed a structured agenda composed of three main steps. First, each group of experts prepared a summary document of current evidence and existing guidelines for its cancer domain, highlighting controversies and unmet needs. Second, during the in-person consensus meeting (2 October 2025), experts presented these summaries and discussed proposed recommendations. Divergent opinions were examined through open discussion moderated by the scientific secretariat. Final statements were reformulated until a unanimous or near-unanimous agreement was achieved. Areas where consensus could not be reached were identified as research priorities or as requiring further evidence before a formal recommendation could be made.

### Literature search and evidence synthesis

A systematic literature review was conducted for each cancer site considered to ensure that the recommendations were grounded in current, robust evidence. Searches were performed in PubMed, Embase, and the Cochrane Library up to July 2025 using combinations of the following keywords: ‘screening’, ‘early detection’, ‘mortality reduction’, ‘risk stratification’, ‘guidelines’, and specific cancer type (e.g. ‘breast cancer screening’, ‘lung cancer Low-Dose Computed Tomography’, etc.).

The inclusion criteria comprised randomized controlled trials, cohort studies, meta-analyses, high-quality systematic reviews, European and international guidelines from professional societies [European Society for Medical Oncology (ESMO), European Society of Breast Imaging (EUSOBI), European Society of Breast Cancer Specialists (EUSOMA), International Association for the Study of Lung Cancer (IASLC), European Association of Urology (EAU), International Agency for Research on Cancer (IARC), and WHO]. Studies were selected if they reported outcomes on mortality, stage distribution, participation rates, cost-effectiveness, or risk stratification models. Evidence was synthesized according to the Grading of Recommendations Assessment, Development and Evaluation (GRADE) principles, and where data were incomplete or heterogeneous, expert interpretation was applied as appropriate (Malmivaara, 2015; Bertolaccini and Rocco, 2018).

### Consensus development and validation

Draft statements were refined following plenary discussions and circulated among all panel members for final review. The writing committee incorporated comments and revisions before approval of the final document. Discrepancies that remained unresolved after discussion were considered open questions for future updates. All sessions were documented, and the final consensus statement was reviewed and endorsed by participants.

### Ethical considerations

As this project involved the synthesis and analysis of expert opinion and published literature, no patient-level data were collected; therefore, ethical committee approval was not required. All contributors adhered to the principles of the Declaration of Helsinki regarding scientific integrity and transparency.

## Results

A concise overview of the principal recommendations and future directions for each tumor type is presented in Table 1.

**Table 1:** Summary of current evidence, recommendations, and future directions for cancer screening according to the European Consensus Project

Cancer type	ECP consensus future directions
Breast	Improve participation through outreach and education; adopt risk-based screening models; integrate artificial intelligence for interpretation and scheduling to enhance efficiency and equity. Integrate primary prevention awareness in the screening environment.
Lung	Implement national, quality-assured LDCT programs integrated with smoking-cessation support; expand eligibility using risk models incorporating genetic, familial, and environmental factors; promote public awareness and adherence.
Colorectal	Offer validated noninvasive options to improve adherence; ensure high-quality colonoscopy and consistent participation across multiple FIT rounds.
Cervical	Integrate vaccination with HPV-based screening and self-sampling; continue evaluation of adjuvant vaccination after conization while maintaining current evidence thresholds.
Prostate	Adopt structured, risk-adapted PSA strategies including mpMRI; de-intensify screening for men with low PSA (<1 ng/ml at 40 years or <2 ng/ml at 60 years); include transgender women aged ≥40 years in tailored protocols.
Gastric	Implement ‘screen-and-treat’ strategies for Helicobacter pylori using noninvasive tests (^13^C-urea breath or stool antigen); focus on early adulthood populations; monitor acceptance through ongoing EUROHELICAN and TOGAS projects.

### Breast cancer screening

Breast cancer remains the most common malignancy among women worldwide. In 2022, 2.3 million new breast cancer cases and 670 000 deaths occurred globally. Annual rates increased in half of the 185 examined countries (Kim *et al*., 2025; Wang *et al*., 2025).

The evidence supporting screening mammography is well established and comes from randomized controlled trials. An IARC working group concluded that women from 50 to 69 years of age who were invited to attend mammographic screening had, on average, a 23% reduction in the risk of death from breast cancer (the information relevant for healthcare policymakers), and women who attended mammographic screening had a greater reduction in risk (Lauby-Secretan *et al*., 2015). The evidence reinforces the notion that screening effectiveness is linked to program regularity, accessibility, and adherence (Al Hasan *et al.*, 2025).

Screening enables earlier diagnosis at a more treatable stage, allowing less aggressive treatments and a better quality of life. The interplay between early diagnosis and treatment, whereby early detection enhances the effectiveness of therapy, remains relevant (Trimboli *et al*., 2020). US data show that in 2023, 5-year relative survival was 99% for localized (stage I) breast cancer and 91% for all stages combined (Siegel *et al.*, 2023). However, in the same country, increasing rates of distant-stage breast cancer at presentation were reported between 2004 and 2021 across all age groups and racial and ethnic categories, from women aged 20–39 to those aged greater than or equal to 75 years, indicating a persistent problem of underdiagnosis (Hendrick and Monticciolo, 2024).

The role of screening mammography within healthcare systems, particularly when balancing benefits and harms, remains an open issue. The UK Independent Panel Review concluded that mammography screening confers an approximate 20% relative reduction in breast cancer mortality among women invited to screening. Still, an 11–19% rate of overdiagnosis, depending on methodological assumptions, was estimated (Independent UK Panel on Breast Cancer Screening, 2012). The following frequencies were estimated: 8 women survived after a breast cancer diagnosis thanks to the screening; 47 survived after a breast cancer diagnosis; 4 had a breast cancer overdiagnosis; 12 died from breast cancer; 30 underwent biopsy of benign findings; 170 were recalled and had further imaging for benign findings; and 729, never recalled, were reassured about the absence of breast cancer (Paci *et al.*, 2014).

Beyond mortality, breast cancer screening also improves prognostic indicators such as tumor size, nodal status, and stage at diagnosis, facilitating breast-conserving surgery and reducing the need for adjuvant chemotherapy. An umbrella review, including 28 meta-analyses, confirmed that, despite heterogeneity in study design, population, and endpoint definition, the direction of the evidence is consistent with a survival benefit (or decreased mortality), albeit of variable magnitude, while highlighting persistent methodological challenges that require transparent risk communication with the public (Shi *et al*., 2025).

Recent guideline harmonization initiatives have sought to reconcile international variability in screening policies. The Italian guidelines, developed using the GRADE-ADOLOPMENT methodology and adopting and contextualizing the European Commission Initiative on Breast Cancer (ECIBC) recommendations, represent a high-quality, evidence-based reference for national programs. It strongly recommends biennial mammography for women aged 50–69 years, conditionally recommends triennial screening for women aged 70–74 years, and recommends annual or biennial screening for women aged 45–49 years, while strongly recommending against routine annual screening after age 50. These guidelines also emphasize the importance of addressing participation inequalities across regions and strengthening program governance and quality assurance (Deandrea *et al*., 2024).

The global synthesis confirmed that most countries maintain mammography as the primary screening tool for women aged 40–74 years, with the greatest impact between 50 and 69 years, and that it advocates more intensive surveillance (including MRI) for women with hereditary or strong family risk factors. While recommendations are largely convergent in high-income settings, the applicability in low-resource contexts remains limited due to infrastructural and workforce constraints (Ren *et al.*, 2022).

Beyond imaging innovation, several proposals address the refinement of screening intervals and population targeting. The trend is shifting from rigid age-based criteria to risk-stratified screening models that incorporate family history, genetic background, hormonal factors, and breast density.

The DENSE study (Bakker *et al*., 2019) pointed out strong evidence from a large randomized controlled trial in favor of using MRI in women with extremely dense breasts (i.e. category *d* according to the BI-RADS classification), showing a reduction in the interval cancer rate of 50% in the intention-to-treat analysis, to 84% in the per-protocol analysis. These results, corroborated by cost-effectiveness analyses (Geuzinge *et al*., 2021), supported the EUSOBI recommendations in 2022 (Saslow *et al*., 2007; Mann *et al*., 2022; Sardanelli *et al.*, 2024). Conversely, despite the availability of RCT evidence supporting MRI screening in women with extremely dense breasts, widespread adoption remains limited, primarily due to organizational constraints.

A systematic review of 21 international guidelines revealed substantial heterogeneity for screening women with dense breasts. While most guidelines recommend continuing mammography (often annually), some endorse the use of digital breast tomosynthesis (DBT) or MRI in selected subgroups. Several guidelines emphasize shared decision-making between clinicians and patients regarding supplemental screening in dense breasts (Al Hasan *et al.*, 2025).

Technological advances, in particular radiomics and deep learning-based artificial intelligence algorithms, are reshaping breast cancer screening paradigms. The MASAI RCT is a milestone in the evaluation of artificial intelligence-assisted mammography reading. Conducted within a public Swedish screening program, it showed a 29% increase in cancer detection rate (6.4 vs. 5.0 per 1000 screened) while reducing radiologists’ reading workload by 44%, without a significant rise in false positives or unnecessary recalls. Most additional cancers detected were small, invasive, and node-negative, suggesting a genuine clinical benefit rather than overdiagnosis (Hernstrom *et al*., 2025). The publication of data from the MASAI trial regarding a 12% reduction in interval cancer rate in the artificial intelligence-supported arm (nonsignificant in the noninferiority design), and of a significant 6.7% increase in sensitivity associated with both arms with a 98.5% specificity, confirms the role of artificial intelligence-supported reading in the future of screening mammography (Gommers *et al*., 2026).

Other studies were in favor of artificial intelligence-supported screening mammography reading, without using randomized study designs with an increase in detection rate (+17%), reduction in reading workflow (−34%), decreased false-positive (−32%), increased positive predictive value of recalls by 48% (Lauritzen *et al.*, 2024), or an increase in detection rate (+18%) and in positive predictive value of recalls (+20%) (Eisemann *et al*., 2025).

In this context, the Italian GRADE-ADOLOPMENT initiative adopted the ECIBC recommendations on artificial intelligence for screening reading but also added the following recommendation now published among the national guidelines system in the website of the Italian Istituto Superiore di Sanità: For asymptomatic women at average risk of breast cancer, the Italian panel suggests using an artificial intelligence-based triage strategy to allocate each mammogram to single or double human reading, instead of performing double human reading for all examinations, in mammography screening based on digital mammography or DBT (Screening e diagnosi del tumore della mammella, 2025).

Artificial intelligence-assisted triage may thus optimize the use of human resources and enable personalized screening intervals based on individual risk and artificial intelligence-derived imaging features. An artificial intelligence-based breast cancer risk prediction model incorporating prior mammograms was validated on over 200 000 women aged 40–74 (Jiang *et al.*, 2025). Using up to 4 years of previous mammogram images, in addition to the most recent mammogram, a 5-year area under the receiver operating characteristic (ROC) curve of 0.78 was obtained based on artificial intelligence analysis of images alone (0.80 for women over 50). These data provide an efficient and effective way to stratify breast cancer risk. Women with a first-degree relative affected by breast cancer may benefit from earlier initiation (often around age 40), whereas carriers of germline pathogenic or likely pathogenic variants (e.g. *BRCA1/2*) typically start annual MRI surveillance from age 25, with mammography/DBT added later (around age 30–35).

The Italian GRADE-ADOLOPMENT approach and recent European initiatives are converging on flexible start-stop ages (45–74 years) and adjustable periodicity, contingent on resource availability and population risk distribution (Ren *et al.*, 2022; Deandrea *et al*., 2024).

In parallel with screening strategies, public health initiatives aim to enhance participation and awareness, particularly among socioeconomically disadvantaged and migrant populations. A review identified key determinants of screening adherence (knowledge, perceived risk, self-efficacy, and cultural beliefs). It advocated educational campaigns, combined with accessible communication and targeted reminders, as essential tools for improving compliance and equity (Tavakoli *et al.*, 2024).

The integration of artificial intelligence technology, individualized risk models, and public education represents the next frontier in maximizing benefits while minimizing harm. Increasing awareness of the need to strengthen primary breast cancer prevention could also be supported by wearable devices and smartphone apps (Sardanelli and Scaperrotta, 2026).

### Lung cancer screening

Lung cancer remains the leading cause of cancer-related mortality worldwide, and its progression can result in late-stage diagnosis that precludes curative treatment (Bertolaccini *et al*., 2025). Early detection through screening has therefore emerged as a pivotal public health intervention capable of substantially reducing mortality. The conceptual foundation of lung cancer screening rests on the observation that localized disease eligible for surgical resection is linked to a markedly better prognosis than advanced stages. The introduction of LDCT enabled the detection of small, asymptomatic nodules at acceptable radiation doses, thereby overcoming the limitations of chest radiography, which historically failed to reduce mortality.

The landmark US National Lung Screening Trial (2011) first established that annual LDCT screening in high-risk populations reduced lung cancer mortality by ~20% compared with chest radiography (Aberle *et al*., 2011). This finding was later corroborated by the Dutch NELSON trial in 2020, which confirmed a significant reduction in lung cancer mortality, particularly among men. The trial demonstrated a more favorable balance between benefits and harms through a volumetric approach to nodule management (de Koning *et al*., 2020). The cumulative evidence from those trials and subsequent meta-analyses indicated that LDCT screening effectively shifts diagnosis toward earlier stages, with a corresponding increase in curative surgical interventions and long-term survival (Passiglia *et al*., 2021).

Current international guidelines have progressively refined the indications for screening. The US Preventive Services Task Force (USPSTF) recommends annual LDCT screening for adults aged 50–80 years with a smoking history of at least 20 pack-years who currently smoke or have quit within the past 15 years (Krist *et al*., 2021). The European position, based on the NELSON results and ongoing implementation studies, supports a similar strategy while emphasizing the need for national programs delivered through quality-assured screening pathways and multidisciplinary teams (Choi *et al*., 2023). The National Comprehensive Cancer Network and other major scientific associations have adopted these recommendations, albeit with some differences regarding cessation criteria, comorbidities, and individualized risk assessment models (Casal-Mourino *et al*., 2021).

Despite these findings, several challenges remain unresolved. Traditional eligibility criteria based solely on age and smoking history may overlook a relevant proportion of lung cancer cases among never-smokers and in populations with environmental or occupational exposures. Risk prediction models incorporating genetic susceptibility, family history, and comorbidities have been proposed to support the selection process (Passiglia *et al*., 2021). Moreover, the appropriate ages for starting and stopping screening are under investigation. Some recent proposals advocate initiating screening at age 45 in specific high-risk subgroups and discontinuing it around age 75 to balance diagnostic yield against the risk of other causes (Choi *et al*., 2023). The duration and frequency of postscreening imaging are also being revisited, with adaptive intervals proposed to minimize radiation exposure and psychological burden without compromising sensitivity.

Future perspectives extend beyond the mere redefinition of risk thresholds. Innovative paradigms support the integration of artificial intelligence to define nodule characterization, perform volumetric growth analysis, and stratify risk. Furthermore, pilot projects are being developed to raise public awareness of the benefits of screening, improve adherence, and ensure equitable access, particularly in underserved regions.

Lung cancer screening represents a paradigm shift in thoracic oncology, moving from a symptom-driven to a preventive approach. The evolving body of evidence establishes LDCT as the key component of early detection in high-risk individuals. The next frontier lies in expanding eligibility through personalized risk modeling, supporting long-term program sustainability through public education, and harmonizing practices within national frameworks that integrate technological innovation with preventive culture.

### Colorectal cancer screening

Colorectal cancer (CRC) is a leading cause of cancer death worldwide, and screening is the single most effective population strategy to reduce both incidence and mortality by detecting advanced precursor lesions and early stage cancers. Over several decades, evidence supporting CRC screening has accumulated from diverse methodological sources: classic randomized controlled trials, pragmatic population studies, nationwide FIT-based program evaluations, and direct comparisons between colonoscopy and serial noninvasive tests. Altogether, these data demonstrate not only that screening prevents CRC and lowers mortality, but also how real-world effectiveness depends critically on program design, test performance, adherence, and the quality of diagnostic colonoscopy.

Early RCTs of guaiac-based fecal occult blood testing showed sustained reductions in CRC mortality at the population level (Hardcastle *et al*., 1996). Similarly, large, long-term trials of once-only flexible sigmoidoscopy demonstrated durable decreases in incidence and mortality that persist for more than a decade after a single screening intervention (Wooldrage *et al.*, 2024). A pragmatic, registry-based randomized trial comparing ‘invitation to colonoscopy’ with usual care in Northern Europe has refined expectations regarding absolute benefits when uptake is modest, reinforcing that program effectiveness depends on both test performance and participation (Bretthauer *et al*., 2022). Complementary evidence from the Iberian multicenter COLONPREV noninferiority trial, which compared an invitation to colonoscopy with an invitation to FIT, has shown that strategies based on high adherence to annual or biennial FIT can achieve mortality reductions at scale, particularly when the quality of diagnostic colonoscopy is ensured (Castells *et al*., 2025). Contemporary nationwide data from FIT-based programs further delineate postcolonoscopy risks and interval cancer dynamics, informing surveillance intervals after a negative colonoscopy (Larsen *et al.*, 2025). In parallel, comparative effectiveness work has quantified the diagnostic yield of single-round colonoscopy vs. once-only sigmoidoscopy and vs. multiple rounds of FIT distributed by mail, situating each along a continuum that balances prevention potential, procedural burden, and adherence (Grobbee *et al*., 2020). In addition, quality-of-care evaluations and professional society recommendations emphasize that endoscopic outcomes (such as adenoma detection rate, withdrawal time, and complication rates) are determinants of the protective effect delivered to individuals and populations (Maida *et al*., 2022).

Current screening recommendations for average-risk adults reflect a growing consensus toward earlier initiation and more individualized discontinuation in older age, accounting for comorbidity and life expectancy. Current leading guidelines recommend that average-risk individuals begin screening at age 45, with a range of valuable options including annual or biennial FIT, multitarget stool DNA (mt-sDNA) testing at defined intervals, colonoscopy at 10-year intervals, and other available tests, and continue through age 75 as a general rule (Knudsen *et al*., 2021). Expert reviews underscore the rationale for starting at 45, given the rising incidence of early onset CRC and favorable benefit–harm profiles at the population level (Issaka *et al.*, 2023; McCabe *et al.*, 2025). Risk-stratified frameworks further refine indications for individuals with a family history, prior advanced neoplasia, or elevated risk phenotypes, including those with one first-degree relative with CRC or advanced adenoma diagnosed before 60 years of age, or two affected first-degree relatives at any age, should start earlier (often at 40 years or 10 years before the youngest diagnosis) and pursue colonoscopy at shorter intervals (Issaka *et al.*, 2023; Ness *et al*., 2024). Organized programs based on FIT adopt posttest pathways that prioritize high-quality diagnostic colonoscopies for positive results, followed by surveillance adjusted according to the findings.

Analyses from mature programs inform the safe extension of intervals after a high-quality negative colonoscopy (Larsen *et al.*, 2025). Across modalities, the central message of current indications is that individuals may choose among multiple validated screening tests, provided that positive, noninvasive results are followed by diagnostic colonoscopy and that endoscopic quality metrics meet or exceed established thresholds (Maida *et al*., 2022; Ness *et al*., 2024; Parodi *et al*., 2024).

New proposals are reshaping colorectal screening along three axes: who should be screened, how screening should be performed, and how often screening should be done. First, on whom to screen, programs are moving toward individualized risk estimation to complement age-based eligibility thresholds. Professional guidance defines risk categories that include family history, prior polyp histology and size, demographic and clinical variables to inform both initiation and surveillance intervals; the absolute value of the FIT test and sex are among the key factors of a personalized approach (Issaka *et al.*, 2023). As the epidemiology of early onset CRC evolves, proposals include earlier outreach to individuals aged 40–44 in high-incidence settings or in those with amplified risk signals (e.g. a strong family history, obesity), coupled with structured shared decision-making for those between 40 and 85 when the expected benefit may still outweigh the harm (Ness *et al*., 2024; McCabe *et al.*, 2025). Second, regarding screening, next-generation noninvasive assays are becoming increasingly validated. A redesigned mt-sDNA test has demonstrated improved sensitivity for CRC and advanced precursor lesions while maintaining acceptable specificity, offering a meaningful advance within stool-based options (Imperiale *et al*., 2024). Blood-based assays using cell-free DNA fragmentation and methylation signatures have achieved sensitivity for CRC and some advanced lesions in average-risk populations, with the critical caveat that any positive blood test must be followed by colonoscopy. That interval performance and cost-effectiveness within organized programs remain under active evaluation (Chung *et al*., 2024). Reviews from multidisciplinary groups contextualize these novel tests as complementary rather than replacements for colonoscopy, particularly given colonoscopy’s uniquely preventive role through polypectomy (McCabe *et al.*, 2025). Among available structural imaging modalities, CT colonography and colon capsule endoscopy continue to serve as alternatives for individuals unwilling or unable to undergo colonoscopy, with program effectiveness depending on the timely completion of diagnostic colonoscopy after positive findings (Ness *et al*., 2024).

Third, regarding how often to screen, proposals increasingly favor dynamic interval assignment based on prior test results, quality metrics, and program-level performance data. In FIT-anchored programs, sustained participation across rounds is the primary driver of population benefit; thus, interventions that improve serial participation (such as simplified logistics, mailed kits, reminders, and navigation) may yield larger reductions in mortality than single-round test sensitivity (Grobbee *et al*., 2020; Castells *et al*., 2025). Nationwide FIT program data showing very low postcolonoscopy CRC incidence after a negative high-quality examination support extension of intervals in appropriate subgroups, freeing resources for outreach and adherence initiatives (Larsen *et al.*, 2025). In colonoscopy-based strategies, adherence to surveillance guidelines prevents both underuse and overuse, the latter of which consumes resources without incremental benefit and increases the risks of procedural harm (Parodi *et al*., 2024). At the health-system level, society’s recommendations emphasize measurable quality standards, including adenoma detection rate benchmarks, and structured feedback loops to ensure uniform program performance (Maida *et al*., 2022; Zheng *et al.*, 2023; Parodi *et al*., 2024).

To sustain and amplify the impact of screening, public health efforts prioritize education and engagement throughout the life course. Campaigns that explain test options, increase acceptance of at-home stool and blood testing, and clarify the need for diagnostic colonoscopy after a positive noninvasive test can improve informed participation, particularly in communities that have been historically under-screened (Issaka *et al.*, 2023; Castells *et al*., 2025). Educational initiatives in schools and communities that improve health literacy on cancer prevention, diet, and physical activity may encourage preventive habits and, in turn, increase screening uptake when people become eligible (McCabe *et al.*, 2025). The integration of patient navigation, multilingual materials, and primary care prompts within organized programs has been repeatedly associated with higher completion rates for both initial testing and diagnostic colonoscopies, thereby translating test performance into effective cancer prevention in practice (Parodi *et al*., 2024; Castells *et al*., 2025). Across these proposals, there is a consistent emphasis on earlier screening initiation in higher-risk populations, broader use of accurate, noninvasive tests, strict attention to colonoscopy quality, and improved adherence and navigation. If implemented consistently, these measures are expected to drive continued declines in CRC incidence and mortality, with benefits more equitably distributed across populations.

### Cervical cancer screening

Cervical cancer screening is one of the major achievements in preventive oncology, having transformed the natural history of a common and lethal malignancy into a largely avoidable disease. Its importance derives from the biological characteristics of cervical carcinogenesis, which unfold over decades through a well defined sequence beginning with persistent infection with oncogenic HPV types, progressing to precancerous intraepithelial neoplasia, and ultimately to invasive carcinoma. This latency period, often spanning 15–20 years, creates a substantial window for detecting and treating precancerous lesions before malignant transformation occurs (Suba *et al.*, 2022).

In settings where organized screening has been established, incidence and mortality from cervical cancer have dropped dramatically (Kyrgiou *et al*., 2020). Conversely, in low- and middle-income countries where such programs remain incomplete or opportunistic, cervical cancer persists as one of the leading causes of female cancer death. These disparities underscore that the impact of screening depends not only on technology but also on population coverage, adherence, and continuity of care.

Over the past two decades, screening paradigms have evolved from morphology-based to molecular approaches. The European Society of Gynaecologic Oncology (ESGO) and the European Federation for Colposcopy state that HPV testing offers superior sensitivity for detecting high-grade lesions, allowing for longer screening intervals without compromising safety (Kyrgiou *et al*., 2020).

The consensus now identifies primary HPV-based screening, with appropriate triage, as the optimal strategy for women aged 30–65 years, replacing cytology as the initial test. Only a limited number of HPV assays meet rigorous clinical validation criteria, and reflex cytology remains the most established triage method. However, HPV (partial/extended) genotyping, p16/Ki67 dual staining, methylation assays, and viral load quantification are under investigation as second-line tests to refine risk stratification (Kyrgiou *et al*., 2020).

In Europe, screening intervals have been progressively extended to five years for women undergoing primary HPV testing, given its high negative predictive value. For younger women, cytology or combined testing is often maintained during the transition to molecular programs between the ages of 25 and 30. An IARC Handbook distinguishes organized population-based programs from opportunistic screening, highlighting that the former achieve superior outcomes through systematic invitation, recall, and quality control procedures (Suba *et al.*, 2022). Audit of all invasive cases remains an essential metric of program performance.

Colposcopy continues to play a pivotal role as the diagnostic and management interface between screening and treatment. The recent European Consensus Statement on Expert Colposcopy redefines standards for complex practice, noting that even as HPV immunization reduces disease prevalence, expert colposcopists will remain indispensable for women at risk (McGee *et al*., 2023). Their contribution ensures accurate diagnosis, avoids overtreatment, and provides the multidisciplinary oversight required for women with atypical cytology, pregnancy, or immunosuppression.

Parallel to these refinements in secondary prevention, the advent of prophylactic HPV vaccination aims to reduce the burden of recurrent disease. Several studies have investigated the roles of adjuvant vaccination, reporting unclear results (Grobbee *et al*., 2020; Maida *et al*., 2022). The VACCIN study (van de Laar *et al*., 2025) and the VITAL study (Caia *et al*., 2026) have examined whether postoperative vaccination after conization reduces the risk of recurrence of high-grade cervical intraepithelial neoplasia, with no short-term benefit observed. The strategy of immunizing women after surgical management to prevent re-infection or viral reactivation remains an area of intense research.

Family history, immunosuppression, and prior high-grade lesions justify individualized surveillance. In parallel, the use of self-collected vaginal samples for HPV DNA testing is emerging as a powerful tool to engage under-screened women, particularly those in remote or socioeconomically disadvantaged areas. Such approaches have demonstrated sensitivity equivalent to clinician-collected samples and can be integrated into national programs through postal or pharmacy-based distribution systems (Kyrgiou *et al*., 2020).

The complete success of screening cannot rely solely on laboratory or clinical advances. As stressed by the IARC and ESGO, public engagement and education are indispensable. Broad-based campaigns must promote awareness from adolescence onward, linking vaccination to later participation in screening. School-based programs, workplace health initiatives, and community outreach can help establish cervical screening as a routine element of adult healthcare, countering stigma and misinformation. Training healthcare professionals to communicate clearly and empathetically with women remains equally critical, especially in multicultural societies where cultural beliefs may hinder participation. In this regard, multidisciplinary collaborations among oncologists, gynecologists, public health experts, and educators are crucial for sustaining adherence and trust.

The evidence synthesized by international bodies converges on a shared framework for comprehensive prevention that integrates vaccination, HPV-based screening, expert colposcopy, and public education.

### Prostate cancer screening

Prostate cancer remains one of the most frequent malignancies and a major cause of cancer-related mortality among men worldwide, making the development of effective early detection strategies a crucial public health goal. The primary objective of screening is to identify clinically significant disease at a curable stage, thereby reducing mortality while minimizing the harms of overdiagnosis and overtreatment. Over the past three decades, large randomized controlled trials have reshaped the scientific landscape, leading to a progressive refinement of screening indications, methodologies, and age thresholds (Andriole *et al*., 2009; Schroder *et al*., 2009; Martin *et al*., 2024; Roobol *et al*., 2025).

The shift from late-stage symptomatic disease toward earlier, potentially curable prostate cancer supports the rationale for screening in populations undergoing systematic testing. The European Randomized Study of Screening for Prostate Cancer (ERSPC), one of the most influential trials in this field, demonstrated that PSA-based screening led to a 20% reduction in prostate cancer–specific mortality compared with unscreened controls after a median follow-up of 9 years (Schroder *et al*., 2009). This landmark study, which included more than 180 000 men across seven European countries, also highlighted that 1410 men needed to be screened and 48 treated to prevent a single death, revealing the delicate balance between clinical benefit and the risk of overdiagnosis. These findings were further supported by long-term analyses from the Rotterdam section of the ERSPC, as well as the most recent trial update at a 23-year follow-up, which indicated that the survival benefit continues to increase with longer follow-up (Roobol *et al*., 2025). After 23 years, the hazard ratio for prostate cancer–specific mortality was 0.87, with an absolute between-group risk reduction for prostate cancer death of 22% (Roobol *et al*., 2025). These results demonstrated that the number needed to invite and the number needed to diagnose to prevent one death fell over time, confirming that the absolute benefit of screening increases with duration.

In contrast, the Prostate, Lung, Colorectal, and Ovarian Cancer Screening Trial failed to show a significant reduction in mortality after 7–10 years of follow-up, mainly due to the contamination of the control group and inadequate differentiation between screened and unscreened populations (Andriole *et al*., 2009).

The issue of screening discontinuation has also been central in the debate. Among men aged 70–74 years who had previously undergone organized PSA screening without a cancer diagnosis, the cumulative risk of prostate cancer mortality by age 85 was only 0.54%, with a dramatic reduction to 0.11% among those with baseline PSA < 2 ng/ml (de *et al.*, 2024). These findings suggest that PSA-based screening may be discontinued in men older than 70 years who have low PSA levels or a history of negative biopsies. Conversely, continued surveillance may be considered in individuals with a PSA level greater than 6.5 ng/ml and a life expectancy exceeding 10 years (Andriole *et al*., 2009).

Further insights emerged from the Cluster Randomized Trial of PSA Testing for Prostate Cancer in the UK, which involved over 400 000 men aged 50–69 years. Although the initial 10-year analysis reported no significant mortality benefit, extended follow-up at 15 years revealed a significant, albeit modest, reduction in prostate cancer–specific mortality (rate ratio = 0.92) among men invited to a single PSA screening compared with standard care (Martin *et al*., 2024). These long-term findings suggest that the benefits of screening, particularly when applied systematically, may take more than a decade to manifest fully, reflecting the slow natural history of prostate cancer progression.

Epidemiological studies conducted after the 2012 USPSTF, recommendation against PSA screening demonstrated a reversal in previously favorable mortality trends. In the period following the USPSTF restriction, men under 75 years exhibited a 1.2-fold increase in cancer-specific mortality and a higher incidence of unfavorable prostate cancer characteristics, namely high PSA, Gleason sum greater than or equal to 8, and high‐risk disease, as well as high clinical N1 proportions. In contrast, no significant effect was observed among men aged 75 years or older (Falkenbach *et al*., 2025). These data confirm that the withdrawal of screening can lead to delayed diagnoses and worse oncological outcomes, reaffirming the necessity for well organized, evidence-based screening programs.

As a consequence, international guidelines have evolved from advocating population-wide screening toward endorsing individualized, risk-adapted strategies. The EAU articulated this paradigm in its 2019 position paper, promoting a structured, personalized approach based on baseline PSA levels, family history, ethnicity, prostate volume, and digital rectal examination, complemented by multiparametric (MRI to refine biopsy indication and reduce overdiagnosis; Gandaglia *et al*., 2019). The EAU recommends offering a baseline PSA test to all well informed men aged 45–50 years with an expected lifespan of at least 10 years, with subsequent screening intervals based on the initial PSA value. This approach reflects a fundamental shift from the binary ‘to screen or not to screen’ to a tailored prevention strategy.

New proposals emphasize the need for refined stratification of screening start and stop ages, as well as tailored surveillance for high-risk subgroups. Men with a strong family history, pathogenic or likely pathogenic germline variants in *BRCA2*, or African ancestry may be considered for earlier screening, typically at 40–45 years of age, within a guideline-based risk stratification framework and with shorter screening intervals (Cornford *et al*., 2024; Martin *et al*., 2024). Those with a baseline PSA less than 1 ng/ml at age 45 can safely extend intervals to 8 years, whereas men with higher PSA levels should undergo testing every 2–4 years. Screening cessation should be considered at age 70–74, particularly for those with low PSA levels and limited life expectancy (de *et al.*, 2024).

An emerging dimension of prostate cancer screening is the inclusion of populations neglected by research and clinical protocols, notably transgender women. Although assigned male at birth, transgender women retain their prostate and therefore remain at risk for prostate cancer, even after gender-affirming surgery. An estimated prostate cancer cumulative incidence of 0.62% among transgender women in the USA has been reported, with age and family history identified as significant risk factors, while gender-affirming hormone therapy (GAHT) appeared to reduce cancer incidence (Martin *et al*., 2024). Importantly, PSA reference values derived from cisgender male populations are inappropriate for transgender women receiving GAHT, as hormonal modulation potentially masks pathological elevations (Nik-Ahd *et al.*, 2023). Subsequent work highlighted challenges in patient identification within electronic records and the urgent need to establish specific clinical and research frameworks for this population (Nik-Ahd *et al.*, 2025). These findings necessitate a reevaluation of screening algorithms, incorporating transgender-specific thresholds and comprehensive patient education to mitigate disparities.

Public health education and awareness campaigns play a vital complementary role in the success of screening. Despite the robust evidence supporting early detection, public understanding of prostate cancer risk and screening implications remains limited. Educational initiatives can promote health literacy and informed consent, ensuring that screening participation is driven by understanding rather than fear or misinformation. Such campaigns should highlight modifiable risk factors, the importance of family history, and the need for regular medical consultation, while integrating modern communication tools and digital platforms to reach diverse populations.

Contemporary evidence converges on the view that structured, risk-adapted PSA-based screening provides a substantial and durable reduction in prostate cancer–specific mortality when applied judiciously. The long-term follow-up of large, randomized trials has dispelled much of the earlier uncertainty, emphasizing that early detection saves lives when balanced against the harms of overdiagnosis and that individualized strategies are most effective. The integration of PSA testing with Multiparametric Magnetic Resonance Imaging, along with targeted robot-assisted prostate biopsy in cases of suspected lesions, genetic and familial risk profiling, and targeted educational initiatives, defines a new era of precision screening. Future directions should aim to expand inclusivity, enhance digital engagement, and embed screening within a broader culture of preventive medicine, transforming prostate cancer detection from a controversial intervention into a refined, equitable, and evidence-based practice that ultimately benefits both individuals and society.

### Gastric cancer screening

Gastric cancer remains a major global health burden, ranking among the leading causes of cancer-related mortality despite a steady decline in several regions. Its prevention and early detection have become pivotal elements of modern gastroenterological practice (Corso *et al.*, 2024). The foundation of contemporary screening strategies is the recognition that *Helicobacter pylori* infection is the dominant etiological factor in chronic gastritis and subsequent gastric carcinogenesis. The Kyoto Global Consensus (Sugano *et al*., 2015) and the Maastricht VI/Florence Consensus (Malfertheiner *et al*., 2022) have established that *H. pylori* is considered an infection-related disease, thereby providing the scientific rationale for population-based eradication and screening initiatives to reduce gastric cancer incidence.

Recent meta-analytic evidence suggests that the global prevalence of *H. pylori* infection has declined in adults from 52.6% before 1990 to 43.9% in 2015–2022; however, prevalence remains relatively high in children and adolescents (Chen *et al*., 2024). This early life infection reservoir highlights the importance of ongoing surveillance and public health strategies, as the decline in *H. pylori* prevalence is correlated with a corresponding decrease in gastric cancer incidence across populations. Nonetheless, substantial geographic heterogeneity is observed, particularly in Asia, Eastern Europe, and Latin America, where prevalence and incidence remain elevated (Park *et al.*, 2025).

From a European perspective, while overall age-standardized incidence rates are relatively low (6.5 per 100 000), striking inter-country disparities remain, with Latvia, Lithuania, and Portugal exceeding 18 per 100 000 (Ferlay *et al.*, 2024). The European Council has therefore recommended implementing gastric cancer screening programs in regions with a high incidence and mortality rate. The ‘screen-and-treat’ strategy, that is, screening for infection in the asymptomatic population to treat infected subjects, emerges as the most feasible approach, especially for younger adults. However, participation of young individuals in the screen-and-treat approach is suboptimal in the recent EUROHELICAN study (Tepes *et al*., 2026). Furthermore, the increased use of antibiotics and the emergence of resistance must be considered (Malfertheiner *et al*., 2022; Leja, 2024).

The 2025 European Society of Gastrointestinal Endoscopy, European Helicobacter and Microbiota Study Group, and European Society of Pathology Management of Precancerous Conditions and Lesions in the Stomach III guidelines reaffirmed the necessity of endoscopic screening in populations with high gastric cancer incidence rates, defined as those exceeding 20 per 100 000 person-years. Screening is not recommended in low-risk countries (age-standardized incidence rate < 10). Furthermore, high-quality endoscopy with virtual chromoendoscopy is endorsed for the detection and staging of atrophy, intestinal metaplasia, and early neoplasia. Surveillance should be individualized based on histologic risk stratification and discontinued after age 80 or when comorbidities outweigh the potential benefits (Dinis-Ribeiro *et al*., 2025).

The Real-world Gastritis Initiative emphasized the critical role of histological identification and staging of gastritis in supporting risk stratification, highlighting *H. pylori* as the primary determinant of the gastric oncogenetic field (Rugge *et al*., 2024). The combined perspective of these consensus efforts reinforces the principle that early diagnosis and eradication of *H. pylori* before the onset of preneoplastic lesions are key to effective secondary prevention (Sugano *et al*., 2015).

An expert group hosted by IARC has analyzed various aspects of the *H. pylori* screen-and-treat strategy for gastric cancer prevention, providing guidance on implementation and suggesting coordinated global efforts to implement this strategy (Park, 2025).

At the population level, Japan and South Korea remain paradigmatic examples of successful organized gastric cancer screening programs that rely on endoscopy and radiographic methods, with demonstrated reductions in mortality (Leja *et al.*, 2014). In contrast, Western nations did not implement national screening programs. However, pilot projects such as GISTAR, TOGAS, and EUROHELICAN are providing necessary evidence for operational feasibility and cost-effectiveness (Leja, 2024).

Over 60% of gastric cancer is attributable to *H. pylori* infection. This staggering proportion supports the inclusion of *H. pylori* screening and treatment programs in national cancer prevention plans, particularly for high-risk regions and younger age groups. The WHO and IARC have both endorsed the exploration of such approaches, emphasizing that up to three-quarters of future gastric cancers could be prevented through targeted eradication (Park *et al.*, 2025).

Future screening frameworks may integrate not only biological risk stratification but also demographic and social factors to provide a comprehensive approach to risk assessment. Family history of gastric cancer, and patients with autoimmune gastritis or extensive intestinal metaplasia represent high-risk subpopulations who warrant more intensive surveillance. Initiating screening at 40–45 years in endemic regions and discontinuing it after 75–80 years may optimize the balance between benefit and cost (Leja, 2024; Dinis-Ribeiro *et al*., 2025).

Artificial intelligence-enhanced endoscopic imaging, already under pilot evaluation, promises to standardize lesion detection and minimize operator dependence (Rugge *et al*., 2024).

Population education remains a cornerstone of any sustainable screening strategy. Public awareness campaigns should target not only adults but also schools, emphasizing *H. pylori* transmission, dietary risk factors (such as high-salt intake and smoking), and the importance of early medical consultation for dyspeptic symptoms.

*Helicobacter pylori* eradication, high-quality endoscopic surveillance, and education-based prevention can collectively reshape the global trajectory of gastric cancer. Public health policies should now move from evidence to implementation, guided by stratified risk models, cost-effectiveness assessments, and coordinated international collaboration.

### Statement of the art on liquid biopsy for cancer screening

Liquid biopsy has emerged as one of the most promising innovations in oncologic diagnostics. Currently, it is employed in disease monitoring, molecular characterization, and assessment of minimal residual disease. Still, it is increasingly being investigated as a potential platform for early cancer detection and population-based screening. Unlike tissue biopsy, which requires direct sampling of the tumor mass, liquid biopsy relies on the analysis of tumor-derived material circulating in bodily fluids, predominantly circulating tumor DNA (ctDNA), circulating tumor cells, and extracellular vesicles. The biological basis of liquid biopsy is that neoplastic lesions shed intact cells and free nucleic acids into the bloodstream. ctDNA released through apoptosis, necrosis, or active secretion may harbor somatic mutations, copy number alterations, epigenetic signatures, methylation patterns, and fragmentomics reflecting the molecular landscape of the originating tumor. The sensitivity of next-generation sequencing, droplet digital PCR, and methylation-specific platforms has increased the ability to detect minute fractions of tumor-associated nucleic acids, enabling the identification of malignant signals even in asymptomatic individuals. The translation of liquid biopsy toward screening paradigms is primarily embodied by the emerging multicancer early detection (MCED) assays. These platforms integrate genomic, epigenomic, and proteomic signals to identify a cancer-specific molecular footprint in peripheral blood and, in some instances, predict tissue of origin. Their principal advantage lies in broad applicability and scalability; a single sample may interrogate for the presence of multiple cancer types, potentially improving detection rates for malignancies lacking routine screening modalities. Such assays are under evaluation in large observational cohorts and controlled trials. Preliminary data suggest that liquid biopsy may complement but not yet replace conventional population screening strategies, such as mammography, colonoscopy, and Pap testing.

Despite rapid technological maturation, several limitations and methodological barriers currently limit the adoption of liquid biopsy for population screening: among others, sensitivity constraints in early stage disease, false positives and indeterminate findings, lack of standardized analytical and preanalytical procedures, regulatory and health-economic hurdles, including demonstration of cost-effectiveness and mortality reduction in large cohorts. These considerations underscore the need for rigorous prospective validation before broad clinical use. As a first step, liquid biopsy could be deployed selectively in individuals with elevated hereditary, environmental, or lifestyle risks. Real-world evidence from ongoing MCED trials will be critical to inform clinical pathways, reimbursement models, and ethical frameworks for implementation.

Liquid biopsy holds the potential to transform cancer screening by offering a noninvasive, repeatable, and system-wide window into tumor biology. While current evidence is strongest for selected tumor types, particularly lung, colorectal, and certain breast and gastrointestinal cancers, further validation is essential to define its role within established screening infrastructures. Its ultimate integration will likely be complementary rather than substitutive.

## Discussion

The ECP consensus process highlights the continued and growing importance of organized, evidence-based cancer screening as a fundamental strategy to reduce cancer morbidity and mortality across the continent. The recommendations presented here derive from a structured expert panel meeting. This approach enabled open, multidisciplinary dialogue and the direct integration of evidence with clinical and public health experience, ensuring that consensus was achieved through shared professional judgment rather than numerical scoring. Such a model, while qualitative, aligns with the established framework for international consensus conferences promoted by the European Commission and the WHO. Precision prevention, as adopted by the ECP, refers to tailoring screening frequency, modality, and intensity according to individual biological, environmental, and behavioral risk factors, rather than relying solely on age. Despite notable successes in established programs for breast, cervical, and colorectal cancers, persistent heterogeneity remained in screening policies, participation rates, and technological adoption among European nations. These disparities often reflect differences in healthcare infrastructure, reimbursement systems, and population awareness, all of which ultimately influence equity in early diagnosis and outcomes. Such heterogeneity underscores the urgency of implementing the 2022 EU Council Recommendation on Cancer Screening (European Council, 2022), which calls for integrated, quality-assured programs across the continent.

Screening should be embedded within comprehensive prevention pathways that integrate smoking cessation, dietary education, and vaccination, thereby ensuring that technological innovation is matched by behavioral and societal engagement. Equity must encompass not only geographic access but also digital literacy, language accessibility, and socioeconomic inclusion across populations.

The panel reaffirmed that screening must remain dynamic and responsive to epidemiological and technological shifts. For instance, the evolution of low-dose computed tomography for lung cancer and HPV testing for cervical cancer exemplifies how innovation can reshape preventive paradigms when guided by robust evidence. Similarly, the integration of digital pathology, artificial intelligence-assisted image interpretation, and PRSs may contribute to refining eligibility criteria, screening intervals, and diagnostic accuracy across tumor types. Nonetheless, implementation of such risk-adapted approaches requires validation within large, population-based frameworks before routine clinical use, as well as careful ethical oversight to prevent the widening of socioeconomic and digital divides.

An additional theme emerging from the consensus was the pivotal role of public-based education in improving adherence. Awareness campaigns and culturally sensitive communication increase participation and acceptance of screening programs. Therefore, national strategies should not only focus on clinical protocols but also embed prevention within broader societal narratives of health literacy, responsibility, and accessibility. Artificial intelligence-driven image interpretation, digital pathology networks, and federated data registries will be key enablers for real-time quality control and adaptive scheduling.

The transition from uniform population screening toward precision prevention is now a realistic goal. Combining lifestyle, environmental, and molecular risk determinants could enable individualized screening schedules that optimize the benefit-to-harm ratio. However, this transition requires the harmonization of data governance, biobanking policies, and ethical standards across the European Union to ensure interoperability and patient trust.

### Limitations

Although the expert panel meeting followed a structured, transparent process, it did not use a formal Delphi or RAND/UCLA scoring system. The recommendations, therefore, reflect shared expert judgment based on the best available evidence rather than quantitative agreement metrics. The strength and maturity of evidence differ across cancer types, being more robust for breast, colorectal, and cervical screening, and comparatively limited for prostate and gastric cancer. The predominance of experts from high-income European regions may also limit the generalizability of the conclusions to settings with fewer screening resources. Furthermore, cost-effectiveness and resource allocation were discussed qualitatively but were not formally analyzed. Given the rapid evolution of imaging, molecular diagnostics, and artificial intelligence integration, periodic updates of these recommendations will be necessary to ensure ongoing validity.

### Conclusion

The ECP Consensus proposes a unified European model of precision screening founded on quality assurance, personalized risk stratification, and digital innovation. By 2030, measurable objectives should include: (a) pan-European indicators for participation and false-positive rates; (b) integration of artificial intelligence-assisted image interpretation in an organized program; and (c) harmonized digital infrastructure for population registries. The ECP Consensus establishes a harmonized European framework for precision cancer screening, integrating artificial intelligence-enabled technologies, personalized risk assessment, and standardized quality assurance. Its implementation aims to ensure that every European citizen can access evidence-based, equitable, and sustainable cancer screening programs. This marks a continental shift from uniform to adaptive prevention paradigms, aligning with Europe’s Beating Cancer Plan.

## Acknowledgements

This work was partially supported by the Italian Ministry of Health with ‘Ricerca Corrente’ and ‘5 × 1000’ funds.

### Conflicts of interest

There are no conflicts of interest.
